# Exploring disparities in the proportion of ultra-processed foods and beverages purchased in grocery stores by US households in 2020

**DOI:** 10.1017/S1368980025000606

**Published:** 2025-04-24

**Authors:** Elizabeth K. Dunford, Donna R. Miles, Barry M. Popkin

**Affiliations:** 1 Food Policy Division, The George Institute for Global Health, University of New South Wales, Sydney, Australia; 2 Carolina Population Center, University of North Carolina at Chapel Hill, Chapel Hill, USA; 3 Department of Nutrition, Gillings Global School of Public Health, University of North Carolina at Chapel Hill, Chapel Hill, USA

**Keywords:** Ultra-processed foods, NOVA classification, Race–ethnic differences, Food environment, Food purchases

## Abstract

**Objective:**

American diets are increasingly based on ultra-processed foods (UPF). Current research, particularly on socio-economic differentials, is lacking. This study aimed to provide an updated examination of US household purchases of UPF and how this differs by race–ethnicity, household income and household education.

**Design:**

The NielsenIQ Consumer Panel 2020 was utilised for analysis. Each food and beverage product purchased by US households was assigned a level of processing under the Nova level of processing classification system. The volume of UPF purchased overall and by food group was determined for each Nova processing group and examined by race–ethnicity, education and income. Results were stratified by race–ethnicity within each income group. A *P* value < 0·0001 was considered significant.

**Setting:**

This study analysed data from the Nielsen IQ Consumer Panel 2020 which recorded household food purchases in the USA.

**Participants:**

The Nielsen IQ Homescan Consumer Panel is a nationally representative longitudinal survey of around 35 000 and 60 000 US households.

**Results:**

Of 33 054 687 products purchased by 59 939 US households in 2020, 48 % of foods and 38 % of beverages were considered UPF. Categories with the highest proportion of purchases deriving from UPF included carbonated soft drinks (90 %), mixed dishes and soups (81 %) and sweets and snacks (71 %). Slightly higher but statistically significant proportions of UPF purchases occurred in the lowest income and education groups and among non-Hispanic whites.

**Conclusions:**

It is concerning that household purchases of UPF in the USA are high. Policies that reduce consumption of UPF may help reduce diet-related health inequalities.

American diets are increasingly based on ultra-processed foods (UPF)^(1,2)^. UPF are defined as ready-to-eat industrial products created from food-derived ingredients combined with food additives through various industrial processes and designed to maximise industry profits. Consumption of UPF is seen to play a part in the global rise in chronic diet-related diseases^(3,4)^ such as CVD, cancer and obesity^(5)^ and has been linked to depressive symptoms^(6)^, sleep problems^(7)^ and even overall mortality^(8)^. In addition to this, research has demonstrated that diets dominated by UPF are nutritionally unbalanced, resulting in a huge influx of studies examining not only the nutritional quality of the American diet, but the level of processing of foods, an element which has been largely ignored in traditional research until recent years^(1)^.

The Nova food processing system is the most widely used approach to classify foods and beverages based on their level of industrial processing^(9–11)^. This system is focused on identifying products that are engineered, manufactured and marketed to promote overconsumption. Nova uses a broad food group-based approach and also considers markers of UPF as coming from twelve Codex classes of additives (flavour enhancers, colours, emulsifiers, emulsifying salts, sweeteners, thickeners and anti-foaming, bulking, carbonating, foaming, gelling and glazing agents) plus flavours and specific ingredients not commonly used at home^(12,13)^. Recent research has shown that using the standard NOVA definition of UPF, around 50 % of food and beverage purchases made by US households in 2020 were considered ultra-processed^(14)^.

Racial/ethnic and income disparities in obesity and nutrition-related chronic diseases among Americans have been well documented. Differences in dietary intake and food purchases across racial/ethnic and income groups may help explain these health inequalities. US-based research using NHANES data between 2007 and 2012 found that those with higher education levels consumed the least UPF, with adolescents, non-Hispanic white and non-Hispanic black ethnic groups the highest consumers^(15)^. The same research also showed that UPF consumption was inversely associated with both age and income levels and did not vary according to sex. Studies using food purchase data, which has benefits over traditional dietary surveys as it captures exactly the specific packaged food products US consumers are buying, found that black and Hispanic households purchased a lower proportion of UPF compared with white households^(16)^. However, this research also found that black households had a striking contrast in purchasing patterns of foods compared with beverages: these households had the highest purchases of highly processed beverages but the lowest purchases of highly processed foods as percentages of calories purchased. Despite these findings, this research is now 10 years old, and the modern food supply is dynamic, with new packaged food products introduced to the market almost daily. At any point in time, there are > 400 000 different packaged food and beverage products available on grocery store shelves.

As such, an up-to-date examination of US food purchasing behaviour may provide unique insight into the persistent disparities in diet quality observed in dietary surveys. There remains a limited understanding of how factors such as household composition, education, income and race/ethnicity intersect to influence consumer food purchasing, particularly when it relates to purchases of UPF. To reduce disparities in the US population, there is a need to understand the types of foods purchased by each population group to better guide policy efforts in the nutrition arena. The objective of this study was to provide an up-to-date examination of US household food and beverage purchases by level of processing under the NOVA classification system. Results were examined overall, by food and beverage category and by demographic subgroups (income, education, race/ethnicity, households with/without children). To ensure clarity, we are not examining the total US diet but rather a nationally representative sample of households and their packaged food purchases from all vendors.

## Methods

### Study design and population

The NielsenIQ Homescan Consumer Panel is used to examine food purchase data for the US population. This panel is an ongoing nationally representative longitudinal survey of between 35 000 and 60 000 households each year and contains information on purchases of packaged food and beverage items at the Universal Product Code level. Households in NielsenIQ Homescan report socio-demographic and household information including gender, income, detailed age composition, education and race (white, black, other) and ethnicity (Hispanic, non-Hispanic) of the head of the household. For the purposes of this analysis, we combined the available race and ethnicity measures to identify when heads of the households were non-Hispanic white, non-Hispanic black, Hispanic or other non-Hispanic. Similarly, data were broken down into three income groups (< 185 % federal poverty level [FPL], 185–350 % FPL, > 350 % FPL), three head of household education groups (high school graduate or less, some college, college graduate or more) and two household types (households with children and households without children). Households included in Homescan are sampled and weighted to be nationally representative. Additional details on this NielsenIQ data can be found in earlier papers^(17,18)^. This article utilised 2020 NielsenIQ Homescan data.

### Linkage of barcodes with nutrition facts panel data

Each uniquely barcoded product captured in Homescan 2020 was linked with nutrition facts label data and ingredient information (where available) using commercial nutrition databases (Gladson, Label Insight, Product Launch Analytics, USDA National Nutrient Database for Standard Reference and Mintel Global New Products Database) for the same time period^(19,20)^. Homescan does not include bulk food items without barcodes, and hence, foods and beverages without a barcode or a nutrition facts label were excluded (e.g. unpackaged fresh fruits and vegetables, fresh meats and loose bread and bakery products without a barcode). It should be noted that these foods are most likely to be consistent with the NOVA definition of minimally processed foods. Based on NielsenIQ modules, products were assigned to nine food categories (fifty-one subcategories) or as a beverage (eleven subcategories).

### Assigning NOVA levels of processing

The literature describing how to determine UPF using the NOVA definition suggests broad food categories that are likely to be UPF^(9,10,21)^. The literature also provides a list of substances never or rarely used in kitchens and twelve Codex classes of additives designed to make the final product palatable as specific markers of UPF. Recent research has also suggested that the use of colours and flavours alone to identify UPF captures 98 % of products considered UPF under the more detailed definition. To this effect, products in each UNC food group were allocated a NOVA ‘group’ from 1–4 (1 = minimally processed foods, 2 = processed culinary ingredients, 3 = processed foods, 4 = UPF). We used the list of substances never or rarely used in the kitchen as well as the twelve Codex additive classes to identify UPF within products that had been broadly classified as UPF under the NOVA food group approach. If none of these substances or additives were found, the food was reallocated to NOVA group 3. Due to differences between Codex and the US FDA’s definitions of additive classes, an iterative approach to align the twelve CODEX classes of additives with the list of industrialised food additives and their technical functions from FDA’s Substances Added to Food inventory, previously known as Everything Added to Foods in the United States (EAFUS)^(22)^, was undertaken which added additives to some of the twelve additive classes highlighted by NOVA (online Supplementary Table 1 and 2).

### Statistical analysis

All analyses were performed using SAS v9.4. The volume of UPF purchased overall, by food and beverage status and by food group was determined for each NOVA processing group. Results were reported overall, by food and beverage and by subcategory for all demographic subgroups. To determine whether associations between purchases and race/ethnicity differed by household income, results were also stratified by race–ethnicity within each income group. Significant differences in the proportion of purchases considered UPF were undertaken using Students *t* test. The tests were done for total foods, total beverages and overall totals of both when examining differences between demographic subgroups. Results for mean volume of foods purchased (in grams or ml) were examined through *t* tests using household projection weights provided by NielsenIQ. Bonferroni multiple comparison adjustment was applied, and a *P* value < 0·0001 was considered significant.

## Results

### Overall results

**Figure 1.**
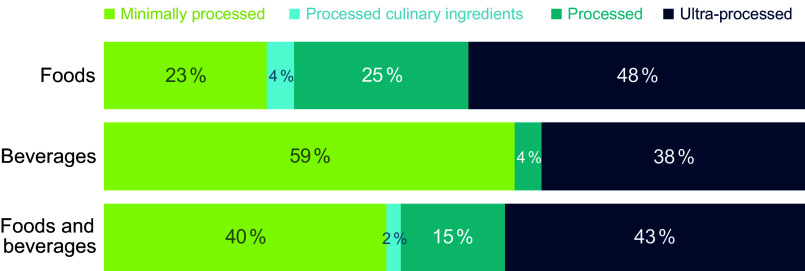
Proportion of food and beverages purchased by US households by level of processing. Footnotes: University of North Carolina calculation is based in part on data reported by NielsenIQ through its Homescan Services for all food categories including beverages for 2020 across the US market. NielsenIQ, 2020^(34)^. Authors’ calculations are based in part on data reported by NielsenIQ through its Homescan Services for all food categories including beverages for 2020 across the US market. NielsenIQ, 2020. The conclusions drawn from the data are those of UNC and do not reflect the views of NielsenIQ. NielsenIQ is not responsible for and had no role in, and was not involved in, analysing and preparing the results reported herein.

**Figure 2.**
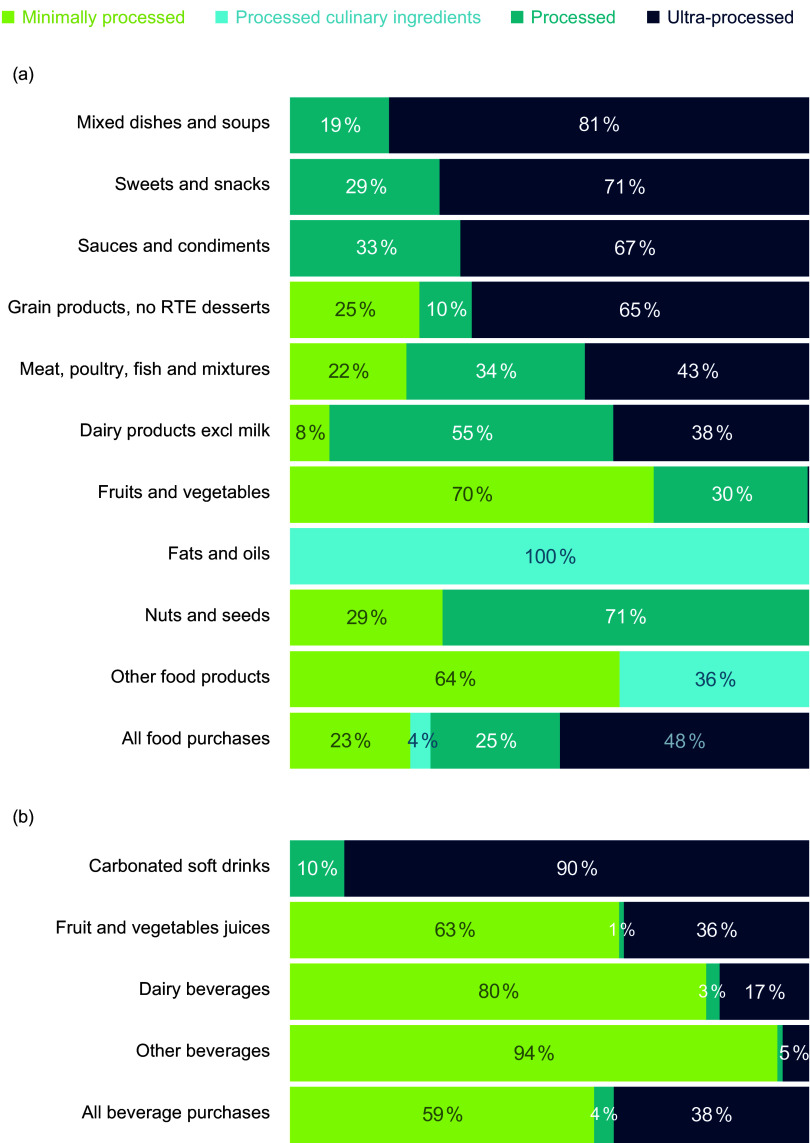
Proportion of (a) foods and (b) beverages purchased by US households by both levels of processing and food category. Footnotes: University of North Carolina calculation is based in part on data reported by NielsenIQ through its Homescan Services for all food categories including beverages for 2020 across the U.S. market. NielsenIQ, 2020^(34)^. Authors’ calculations are based in part on data reported by NielsenIQ through its Homescan Services for all food categories including beverages for 2020 across the US market. NielsenIQ, 2020. The conclusions drawn from the data are those of UNC and do not reflect the views of NielsenIQ. NielsenIQ is not responsible for and had no role in, and was not involved in, analysing and preparing the results reported herein.

Characteristics of the study population are presented in Table 1. Out of 33 054 687 products purchased by 59 939 US households in 2020, 43 % of packaged food and beverage products were considered ultra-processed (NOVA group 4; 48 % foods and 38 % beverages; Figure 1). The categories with the highest proportion of purchases deriving from UPF included carbonated soft drinks (90 %), mixed dishes and soups (81 %) and sweets and snacks (71 %) (Figure 2). Fruits and vegetables, fats and oils and nuts and seeds all had 0 % of purchases considered ultra-processed. These results were further reflected when examining which categories represented the largest proportion of US household purchases of UPF (online Supplementary Figure 1), with carbonated soft drinks representing the largest proportion of UPF purchases (28 %), followed by sweets and snacks (20 %) and mixed dishes and soups (13 %). Importantly, although categories such as fats and oils, nuts and seeds and fruits and vegetables had zero purchases deriving from UPF, these categories made up only a small fraction of overall purchases (12 %; Table 2) compared with the top three categories contributing to UPF purchases which made up 36 % of total purchases.

**Table 1.** Demographic characteristics for NielsenIQ^*^ 2020 household panel (*n* 59 938)

Characterisitic^†^	%	*n*
Household size		2·5
Any children	30·7 %	12 975
Race/ethnicity of household head		
Non-Hispanic white	66·9 %	44 872
Hispanic	13·8 %	4499
Non-Hispanic black	11·7 %	6694
Non-Hispanic other	7·5 %	3873
Educational attainment (highest in household)		
High school or less	2·1 %	588
High school graduate	24·1 %	9753
Some college	30·9 %	16 543
College graduate	27·2 %	21 243
Postcollege graduate	15·6 %	11 811
Household income		
< 185 % FPL	26·8 %	12 516
185–350 % FPL	28·2 %	20 013
> 350 % FPL	45·0 %	27 409
Observations		59 938

FPL, federal poverty level.

* University of North Carolina at Chapel Hill calculations based in part on data reported by NielsenIQ through its Homescan Services for all food categories, including beverages and alcohol for 2020 across the US market (NielsenIQ, 2021). The conclusions drawn from the data are those of UNC and do not reflect the views of NielsenIQ. NielsenIQ is not responsible for and had no role in, and was not involved in, analysing and preparing the results reported herein.

† Calculations utilised projection weights provided by NielsenIQ which take into account the following factors: household size, income, household head age, race–ethnicity, education, occupation, presence of children and county size.

**Table 2.** Proportion of volume sales deriving from each food category

Category	Proportion of volume sales
Food	52 %
Dairy products excluding milk	5 %
Meat, poultry, fish and mixtures	3 %
Nuts and seeds	1 %
Grain products, no RTE desserts	8 %
Fruits and vegetables	10 %
Fats and oils	1 %
Sauces and condiments	3 %
Sweets and snacks	11 %
Other	2 %
Mixed dishes and soups	7 %
Beverages	48 %
Dairy beverages	11 %
Fruit and vegetable juices	8 %
Carbonated soft drinks	16 %
Other beverages	15 %

RTE, ready to eat.

### Demographic subgroup differences

**Figure 3.**
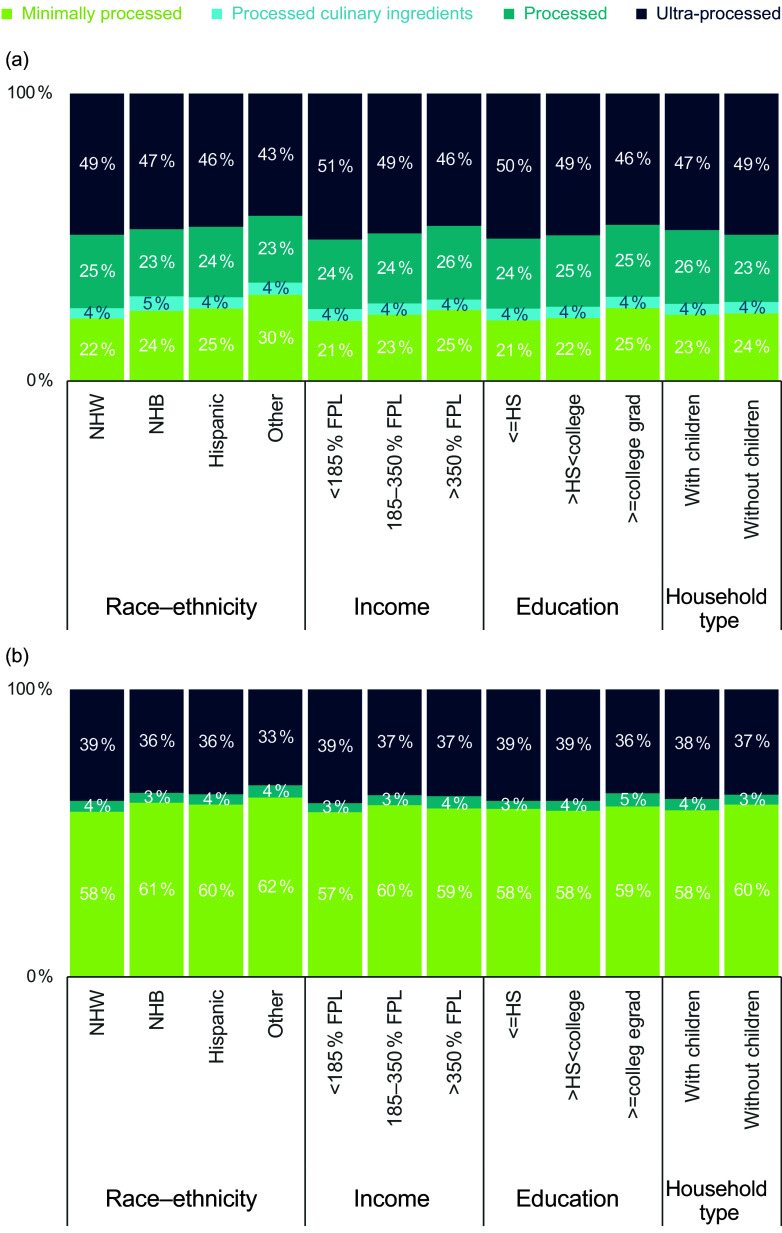
Proportion of beverages (a) and foods (b) purchased by US households by level of processing, by demographic subgroup. Footnotes: University of North Carolina calculation is based in part on data reported by NielsenIQ through its Homescan Services for all food categories including beverages for 2020 across the US market. NielsenIQ, 2020^(34)^. Authors’ calculations are based in part on data reported by NielsenIQ through its Homescan Services for all food categories including beverages for 2020 across the US market. NielsenIQ, 2020. The conclusions drawn from the data are those of UNC and do not reflect the views of NielsenIQ. NielsenIQ is not responsible for and had no role in, and was not involved in, analysing and preparing the results reported herein.

We observed small but statistically significant differences across the various socio-economic subgroups. Non-Hispanic white households purchased a significantly higher proportion of UPF (49 %) compared with non-Hispanic black households (47 %; *P* < 0·0001), Hispanic households (46 %; *P* < 0·0001) and other race–ethnicity (43 %; *P* < 0·0001) (Figure 3(a)). The same race–ethnic trends were seen for beverages (Figure 3(b)). For food, significant differences in the proportion of purchases considered UPF were seen between all income groups, education groups and between households with and without children (*P* < 0·0001 for all; Figure 3(a)). The lowest income group (< 185 % FPL) and lowest education group (≤ high school) had the highest proportion of food purchases deriving from UPF (51 % and 50 %, respectively), with the highest income group (> 350 % FPL) and highest education group (≥ college degree) having the lowest proportion. For beverages, smaller but still significant differences were observed showing the same trends (Figure 3(b)).

When results were stratified by race–ethnicity and income, the same trends were observed, with higher income groups across all race–ethnic groups having a lower proportion of food and beverage purchases deriving from UPF (online Supplementary Figure 2). Overall trends persisted when comparing race–ethnic results within the same income group. For example, 52 % of food purchases made by non-Hispanic white low-income households were considered UPF, significantly higher than low-income non-Hispanic Black, Hispanic and other race–ethnicities.

Significant food group results included that non-Hispanic whites had significantly higher proportions of purchases deriving from UPF compared with all other race–ethnic groups in carbonated soft drinks, and non-Hispanic blacks had higher proportions from fruit and vegetable juices, and dairy beverages (*P* < 0·0001; online Supplementary Figure 3). The other race–ethnicity group had the lowest proportion of purchases deriving from UPF in all food and beverage groups except dairy beverages. Grain products and fruit and vegetable juices had the most varied results, with at least a 10 % difference between the lowest and highest proportions of purchases considered UPF.

## Discussion

There is consensus that UPF in the US diet are increasing^(2)^. To the knowledge of the authors, this analysis is the most current examination of demographic differences in US household purchases of UPF. In this nationally representative sample of US households, we found that UPF dominated overall purchasing patterns by collectively representing 48 % of barcoded packaged food purchases and 38 % of beverage purchases. The most recent published research from 2012 examined the proportion of food and beverage purchases deriving from UPF and found that 52 % of energy purchased derived from processed and UPF, indicating that not much change has occurred in the past 11 years^(23)^. Research using NHANES data from 2018 has estimated UPF consumption higher than what we observed using purchase data; with the latest data indicating 57 % of foods consumed by the US population derived from UPF^(2)^. UPF also dominate the diet in many other Western countries. In the UK, UPF are estimated to represent 57 % of dietary intake^(24)^, 42 % in Australia^(25)^ and 48 % in Canada^(26)^.

Non-Hispanic whites had a significantly higher proportion of purchases of packaged foods and beverages deriving from UPF compared with all other race–ethnic groups. These overall results align for the most part with existing research, although with some notable differences. Although earlier research from 2012 showed that non-Hispanic white households purchase a higher proportion of UPF, it also found that non-Hispanic blacks purchased a significantly higher proportion of ultra-processed beverages compared with other race–ethnic groups^(16)^. This study did not observe this difference, finding that non-Hispanic white households in 2020 purchased a larger proportion of ultra-processed beverage products. A number of reasons may explain this difference in observations in this study. Firstly, although non-Hispanic white households had a higher proportion of carbonated soft drink purchases considered UPF, non-Hispanic black households had a higher proportion of fruit and vegetable juices and dairy beverages considered UPF. Secondly, this study examines household purchases and does not include UPF beverages that may be consumed outside the home. Data from the U.S. Department of Agriculture National Food Acquisition and Purchase Survey have previously shown that non-Hispanic blacks are more likely than other race–ethnic groups to purchase SSB outside the home^(27)^. Thirdly, the dataset for this study was obtained during the first year of the COVID-19 pandemic, and as such may have led to differences in food purchasing behaviours. Research for example has shown that racial/ethnic minorities were more likely to be food insecure during the pandemic^(28)^, which might explain why non-Hispanic whites had a higher proportion of UPF purchases compared with other groups during this timeframe. It will be critical to examine data from the years following the COVID-19 pandemic to truly know for sure what factors led to the results found in this study.

In our sample, the lowest income group (< 185 % FPL) and lowest education group (≤ high school) had the highest proportion of packaged food purchases deriving from UPF, with the highest income group (> 350 % FPL) and highest education group (≥ college degree) having the lowest proportion. This supports previous research that has demonstrated the lower income groups consume a higher proportion of UPF compared with higher income groups^(16)^ and is in line with research showing that high-income households purchase more healthy fruits and vegetables and fewer unhealthy processed meats and SSB compared with low- and middle-income households^(29)^.

Considering the growing body of research linking inadequate intake of important nutrients and risk of chronic diseases, and the high proportion of household purchases that are considered UPF, these findings have implications for policy and practice. Currently, policy approaches in the USA do not consider the level of food processing. Emerging research is showing that the combination of nutrient profiling with an examination of the level of processing is the most useful method to identify less healthy food and beverage products^(14)^. The NOVA classification system can be relatively complex to apply, limiting its accuracy and use for policymakers looking to address the more than 400 000 food and beverage products on grocery store shelves in the USA. At a minimum, initiatives that combine the promotion of healthy foods with the reduction of UPF consumption should be considered^(30)^.

### Strengths and limitations

A strength of this study was the use of nationally representative food purchase data collected over an entire year. A limitation of this research is that household purchase data is only a proxy for consumption and does not equate to total dietary intake as products purchased and consumed outside of the home are not included. Homescan also does not capture purchases from fast-food chains and other restaurants, which means that the proportion of UPF purchased in the present study is likely largely underestimated. A limitation of this study is that households in Homescan do not report whether all food and beverage purchases were consumed, and hence, the amount of food waste is unknown. Another limitation is that food products without barcodes cannot be scanned and linked to purchases, so these items were excluded from analyses. The current literature also reports ambiguity in how to apply the Nova classification system, which is known to impact how accurately and reliably Nova is applied^(31)^. In this study, UPF were identified using product ingredient lists to identify UPF markers as well as specific food group allocation, which aligns with the recommended approach from previous observational studies linking UPF consumption to negative health outcomes^(10)^. Although misreporting is possible, the accuracy of the NielsenIQ data is based on scans of household food purchases so expected to be quite accurate. As discussed, the fact that data were collected during the COVID-19 pandemic is also a potential limitation in that it may not be as representative of consumer purchases compared with other years. In fact with reduced eating-away-from-home, grocery purchases increased, as did unhealthy package food purchases^(32,33)^.

### Conclusion

With USA dietary guidelines recommending consumers focus on meeting food group needs with nutrient-dense foods and beverages, it’s concerning that a large proportion of household purchases of packaged foods and beverages derive from UPF. We found that 43 % of purchases of barcoded packaged foods and beverages from grocery stores made by US households in 2020 were derived from UPF. The proportion could be even higher if considering restaurant and take-out foods. This highlights the need for policy action to increase the healthiness of the broader food environment and subsequently positively impact obesity and NCD rates, particularly seeing as the USA is one of the only countries in the Americas without any federal regulations to reduce intake of UPF, despite the huge growing literature from birth to death of the impact of UPF on a vast array of biological mechanisms and health outcomes.

## Supporting information

Dunford et al. supplementary material Dunford et al. supplementary material

## Data Availability

Data described in the manuscript, code book and analytic code will not be made available due to a licensing agreement with NielsenIQ.

## Supplementary material

For supplementary material accompanying this paper visit https://doi.org/10.1017/S1368980025000606
