# Supplementary material for: Exploring disparities in the proportion of ultra-processed foods and beverages purchased in grocery stores by US households in 2020
Source: Public Health Nutr. 2025 Apr 24;28(1):e85. doi: 10.1017/S1368980025000606 (PMC12100555; doi:10.1017/S1368980025000606)

Exploring the proportion of ultra-processed foods and beverages purchased by US households in 2020 — **Supplementary material**

Supplementary Table 1: Ingredient search terms specified under NOVA to identify UPFs

**Term used to search ingredient list**
Hydrolyzed protein
Soya/soy protein isolate
Casein
Whey protein
Mechanically separated meat
Invert sugar
Invert sugar syrup
Maltodextrin
Dextrose
Soluble Fiber
Insoluble Fiber
Hydrogenated Oil
Interesterified Oil
Fructose
Lactose
Gluten
Fruit Juice Concentrate

Supplementary Table 2: Mapping of CODEX Functional Additive Classes to FDA Technical Effect Classes

UPF Additive Category	CODEX Functional Class	FDA Technical Effect
Anti-foaming agent	Anti-foaming agent	Surface-active agents
Foaming agent	Foaming agent	
Bulking agent	Bulking agent	Stabilizers and thickeners
Gelling agent	Gelling agent	
Thickener	Thickener	
Carbonating agent	Carbonating agent	Propellants, aerating agents, and gases
Colour	Colour	Color and coloring adjuncts
Emulsifier	Emulsifier	Emulsifiers and emulisifier salts
Emulsifying salt	Emulsifying salt	
Flavour	*<NA>*	Flavoring agents and adjuvants
Flavour enhancer	Flavour enhancer	Flavor enhancers
Glazing agent	Glazing agent	Surface-finishing
Sweetener	Sweetener	Non-nutritive sweeteners

SUPPLEMENTARY FIGURE 1

Categories contributing to UPF purchases

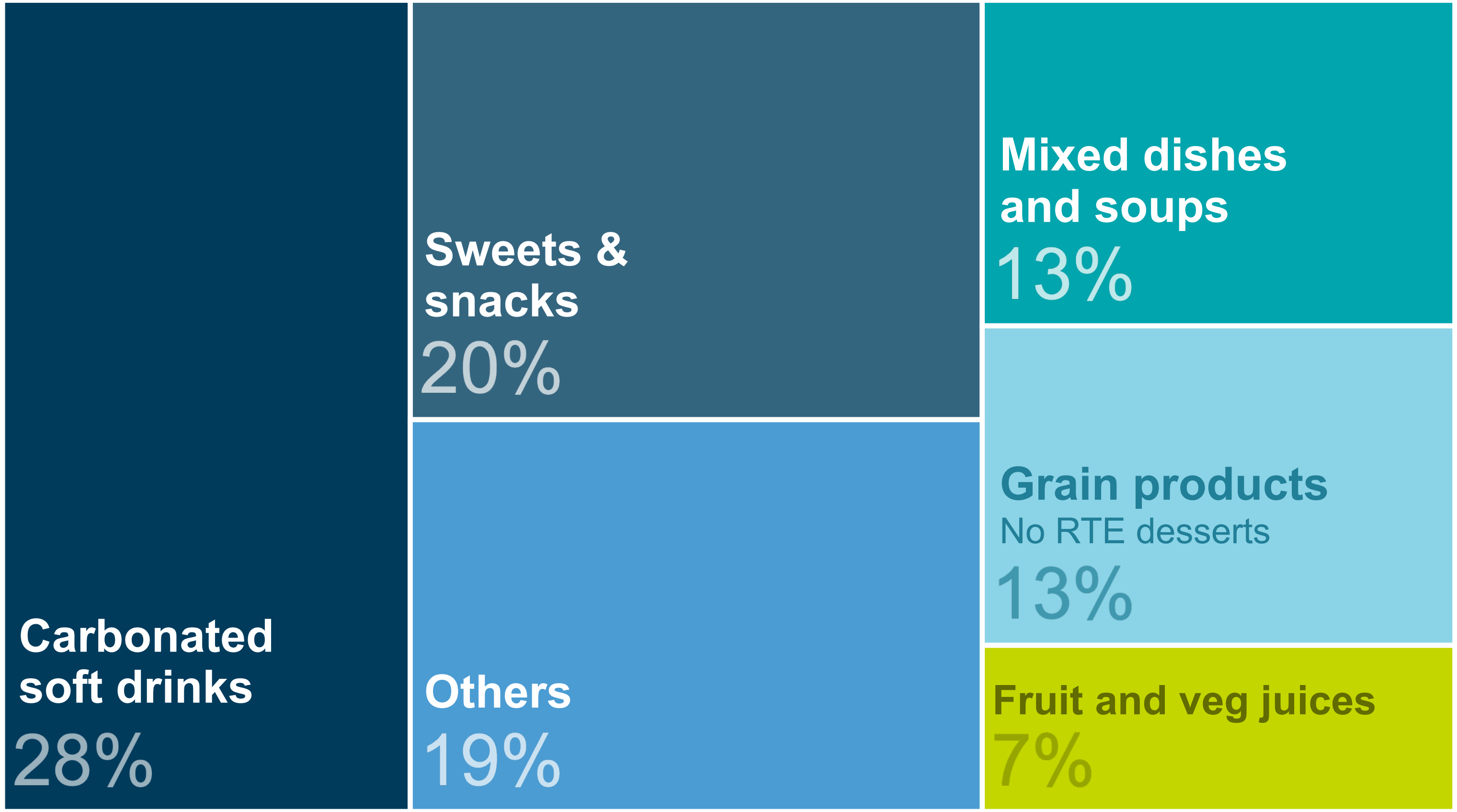

SUPPLEMENTARY FIGURE 2

Proportion of food (A) and beverage (B) purchases in each level of processing group, stratified by race-ethnicity and income

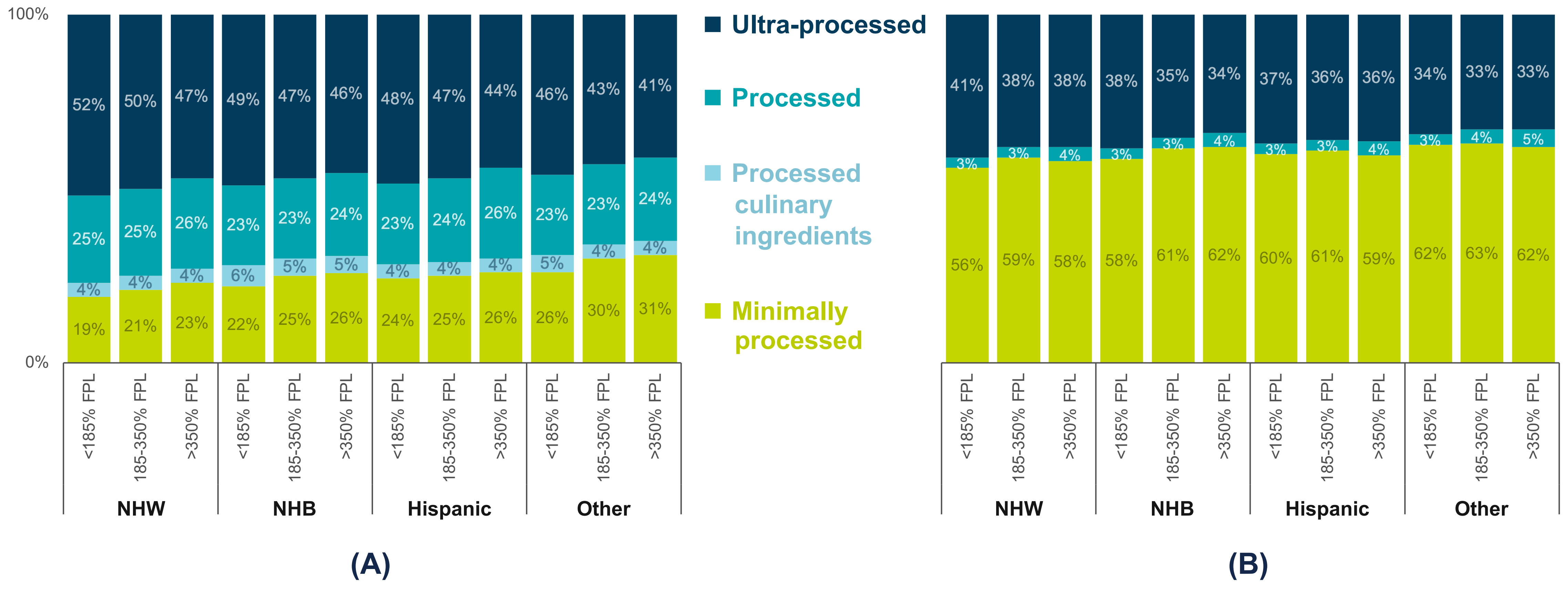

SUPPLEMENTARY FIGURE 3

Proportion of food and beverage purchases deriving from UPFs in each category, by race-ethnicity

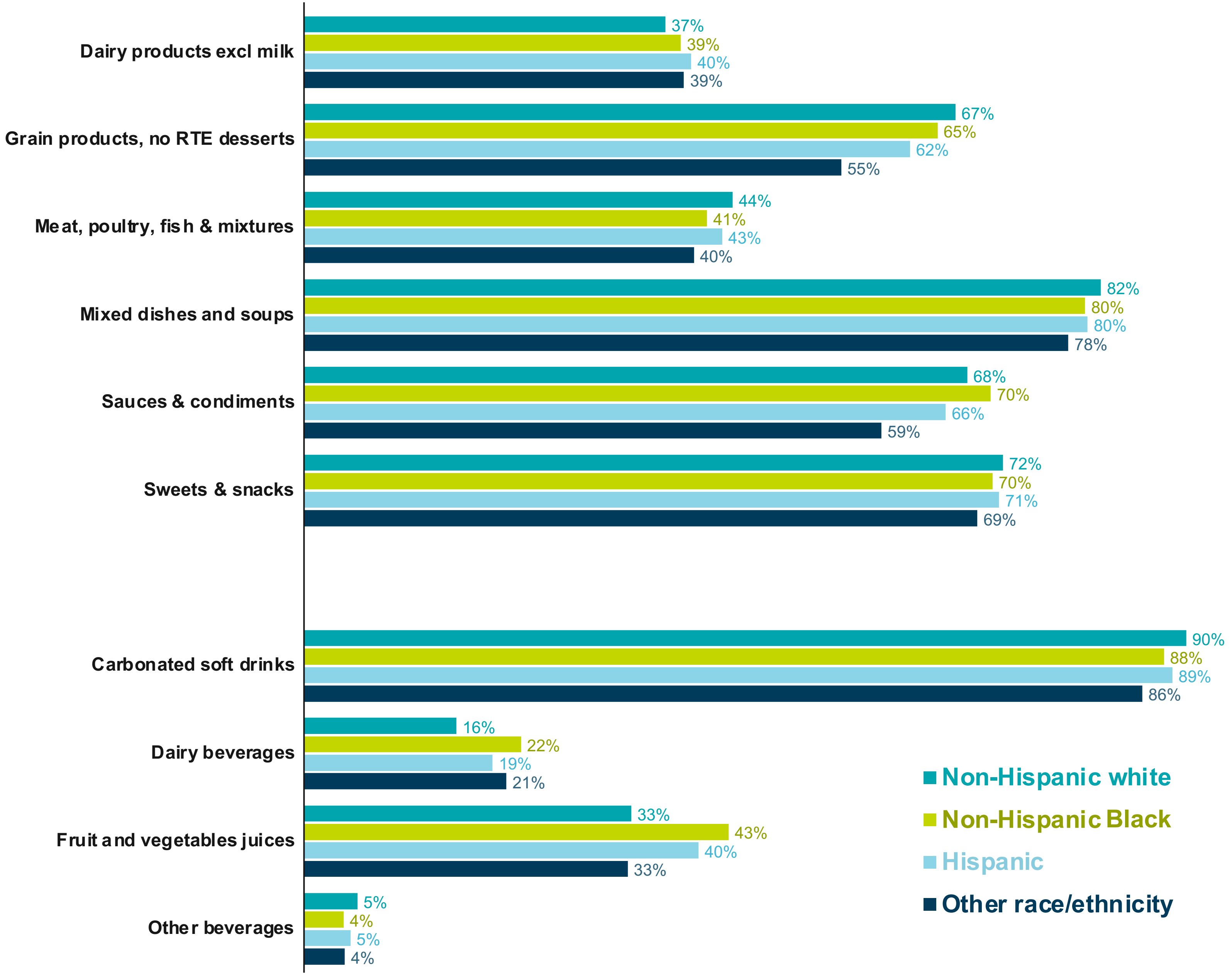

Supplement: Dunford et al. supplementary material [file S1368980025000606sup001.pdf]
